# Neat Chitosan Porous Materials: A Review of Preparation, Structure Characterization and Application

**DOI:** 10.3390/ijms23179932

**Published:** 2022-09-01

**Authors:** Paweł Grzybek, Łukasz Jakubski, Gabriela Dudek

**Affiliations:** Department of Physical Chemistry and Technology of Polymers, Faculty of Chemistry, Silesian University of Technology, Strzody 9, 44-100 Gliwice, Poland

**Keywords:** porous chitosan material, phase inversion, sol-gel, cryogelation, porogen agent, freeze-drying

## Abstract

This review presents an overview of methods for preparing chitosan-derived porous materials and discusses their potential applications. This family of materials has garnered significant attention owing to their biocompatibility, nontoxicity, antibacterial properties, and biodegradability, which make them advantageous in a wide range of applications. Although individual porous chitosan-based materials have been widely discussed in the literature, a summary of all available methods for preparing materials based on pure chitosan, along with their structural characterization and potential applications, has not yet been presented. This review discusses five strategies for fabricating porous chitosan materials, i.e., cryogelation, freeze-drying, sol-gel, phase inversion, and extraction of a porogen agent. Each approach is described in detail with examples related to the preparation of chitosan materials. The influence of the fabrication method on the structure of the obtained material is also highlighted herein. Finally, we discuss the potential applications of the considered materials.

## 1. Introduction

**Figure 1:**
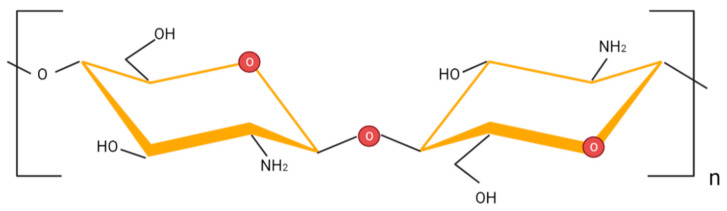
Chemical structure of chitosan.

Chitosan is one of the most popular polysaccharides, with applications in many areas. Similar to other macromolecular polysaccharides, chitosan chains comprise numerous basic units connected by glycosidic bonds. Specifically, chitosan is a linear copolymer consisting of 2-acetamido-2-deoxy-β-D-glucopyranose and 2-amino-2-deoxy-β-D-d-glucopyranose, linked in a β-1,4-configuration [1]. The chemical structure of chitosan is shown in Figure 1.

Chitosan is obtained following the deacetylation of chitin, which is found primarily in crustaceans, mollusks, insects, and fungi. The main sources of industrial chitosan are shrimp and crab waste. In these cases, chitin is a component of the crustaceans’ exoskeletons, and its content in dried exoskeletons varies from 5% to 42%, depending on the species [2,3]. Mushrooms and related wastes after industrial processing are also valuable sources of chitosan, especially because the chitin content can reach 50% in relation to the dry biomass. Chitin is also found in the spores and cell walls of hyphae, where it forms microfibrils in combination with glucan molecules. Chitosan is furthermore an important structural component of cell walls in some species of fungi, such as *Mucor*, *Absidia*, and *Rhizopus*. One particularly important class is Zygomycetes, wherein the *Gongronella butleri* species gives the highest yield of chitosan [4,5,6]. Alternative sources of chitosan include insects and mollusks; however, these have only been used on a laboratory scale, until recently. The literature reports indicate the potential for extracting chitosan from insects, such as beetles, blowflies, silkworm chrysalides, and houseflies, as well as mollusk species, such as *Sepia kobiensis*, *Loligo lessoniana,* and *Loligo formosana*. In these cases, the amount of retrievable chitosan and its degree of deacetylation can vary depending on the animal’s living environment [7,8].

**Figure 2:**
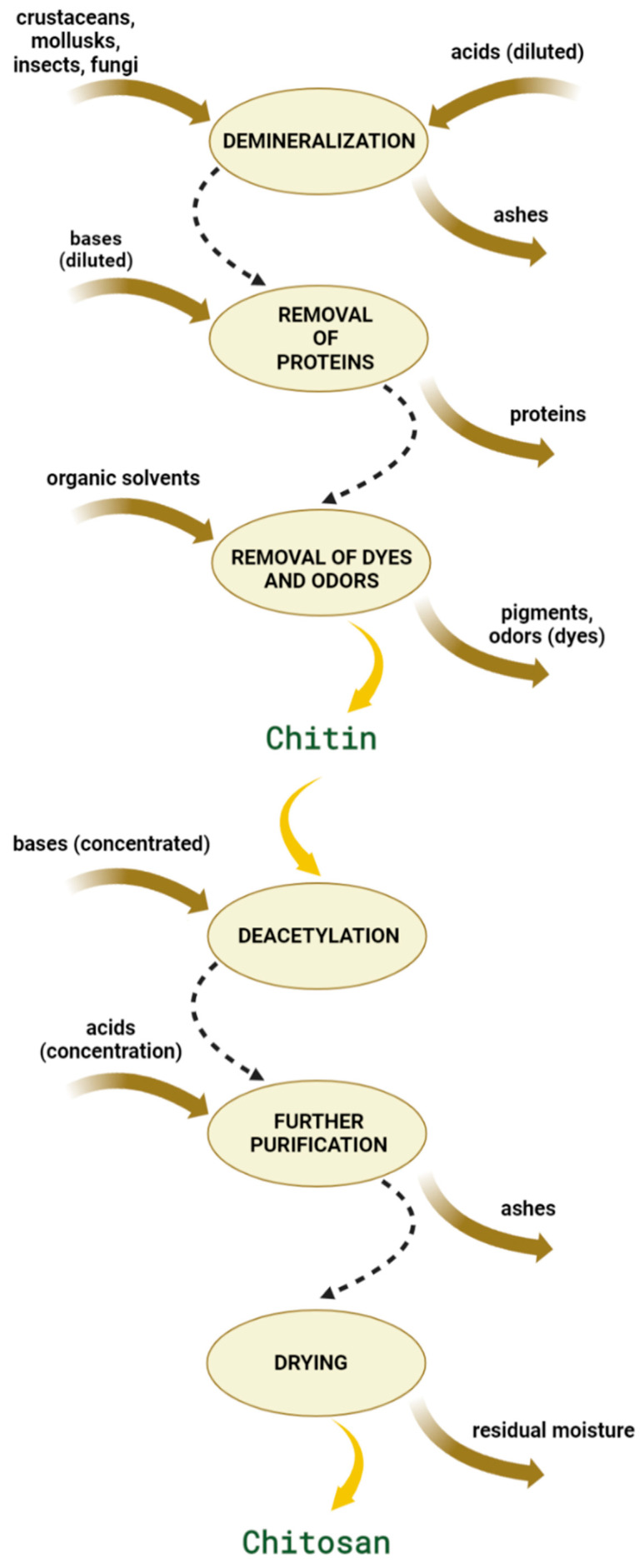
Scheme showing the production of chitosan from chitin using the conventional method.

Chitosan is typically obtained from chitin using one of two possible methods: the conventional extraction, wherein chitin is extracted from crustaceans, insects, or mollusks, or the fermentation process, wherein chitosan is obtained from fungi [2]. In the conventional process, chitin is isolated from the source in a few steps. First, the raw materials are treated with diluted acid (i.e., HCl or HNO_3_) to promote demineralization. This procedure removes mineral contents in form of inorganic salts (ashes) without destroying the chitin structure or inducing depolymerization. In the second step, proteins are removed either with a dilute base (i.e., 10% NaOH solution) or via enzymatic reactions with tripsine [9]. The chitin is then isolated from the reaction mixture by removing other organic compounds. In the next step, chitin is converted into chitosan through alkaline deacetylation, whereby acetamido groups are converted to amino groups. Deacetylation occurs in strong alkali environments and at elevated temperatures (i.e., 80–120 °C). These two parameters (pH and temperature) have the most significant impact on the physico-chemical properties of the obtained chitosan. To purify the isolated product from other solid precipitates, it can be dissolved in an acidic medium, centrifuged, and then filtered. In the last step, chitosan is dried to ensure a sustainable degree of moisture in the final product [2]. A scheme illustrating the conventional method used to obtain chitosan from chitin is presented in Figure 2.

In contrast, the fermentation process yields fungal biomass containing glucan complexes with chitin or chitosan [4,5,6]. Two types of fermentation can be used to obtain chitin and chitosan from fungi: solid-state fermentation, wherein microbial growth occurs on moist solid media with no free-flowing water, and submerged fermentation, which takes place in liquid medium. The additional steps for obtaining chitosan from chitin are the same as those described for the conventional extraction method.

The properties of chitosan depend on its molecular weight and degree of deacetylation [10,11,12,13]. A characteristic feature of chitosan is its abundance of hydroxyl (–OH) and amine groups (–NH_2_). The important properties of chitosan that govern its applicability include solubility, polycationic character, film-forming abilities, antimicrobial properties, and biocompatibility. The solubility and polycationic character of chitosan are fundamental properties that impact the preparation of derivatives and applications in several fields. Complete chitosan dissolution is only possible in a slightly acidic medium because of the protonation of –NH_2_ groups. The presence of ammonium groups (–NH_3_^+^) generates strong electrostatic repulsions between the polymer and surrounding solvent molecules, which consequently improves the solubility of chitosan. On the other hand, strong interactions between positively charged ammonium groups and negatively charged entities (i.e., proteins or lipids) make chitosan an attractive polymer for stabilizing chitosan-DNA fragment complexes [14,15,16]. Such interactions with negatively charged molecules are also important when introducing chitosan as a component of diet supplements that bind free fatty acids. During the digestion process, lipids are released, and therefore, the presence of chitosan in diet supplements facilitates the removal of harmful lipid-based substances [17,18,19]. Furthermore, in acidic environments, chitosan behaves as a cationic polyelectrolyte, which enables ionic modifications that endow chitosan with amphiphilic properties [20,21]. Amphiphilic chitosan has been widely studied in drug delivery systems for its potential to protect encapsulated drugs from harsh conditions [22,23,24], increase the uptake of targeted cells [25], improve mucoadhesive properties [26], and increase the bioavailability of drugs [27].

A key property of chitosan, which makes it attractive for application in food preservation, pharmaceuticals, and biomedicine, is its antibacterial and antifungal effects. Two mechanisms have been proposed to explain the antibacterial and antifungal properties of chitosan. The first pathway assumes that positively charged groups in chitosan chains (i.e., amine groups) interact with negatively charged particles that are present at the cell surface, where the chitosan is responsible for binding and destroying pathogens. The second mechanism is based on the formation of chitosan-DNA complexes. In this case, free particles of chitosan permeate into the cell nucleus where they react with DNA to inhibit mRNA synthesis, thereby preventing the creation of pathogen proteins [14,15,16,28].

Another important property of chitosan originating from the presence of –OH and –NH_2_ groups is its ability to chemically absorb metal and dye ions. Two chemical adsorption mechanisms can be distinguished based on the type of substance absorbed by the chitosan and the pH of the medium: chelation and electrostatic attraction. In the case of chelation, ions interact with amine or hydroxyl groups through covalent bonds, resulting in complex formation. The second mechanism involves the electrostatic attraction between chitosan chains and negatively charged particles [29,30,31,32]. 

One of the most important properties of chitosan is its ability to form films owing to its inert, hydrophilic nature and its insolubility in water, as well as alkali and organic solvents. These properties allow for the formation of solid/gel films or membranes with interesting mechanical, chemical, and textural properties. Such chitosan films and membranes have been used for the separation of gas and liquid mixtures (i.e., ultrafiltration, reverse osmosis, and pervaporation) and as biodegradable films in the food packaging industry. There are numerous examples of chitosan being employed as a selective membrane to separate different types of liquid mixtures. Because of its hydrophilic nature and affinity toward water, chitosan membranes have been intensively studied for the dehydration of alcohols. Studies regarding the separation of organic mixtures with chitosan membranes are also abundant [33,34,35,36,37]. Chitosan films have been investigated for food packaging applications owing to their favorable mechanical properties and selective permeability to CO_2_ and O_2_. Furthermore, their excellent antibacterial and fungicidal properties, biocompatibility, nontoxicity, and ease of film forming make them attractive edible packaging materials that can improve food safety and increase shelf life [38,39,40,41].

**Figure 3:**
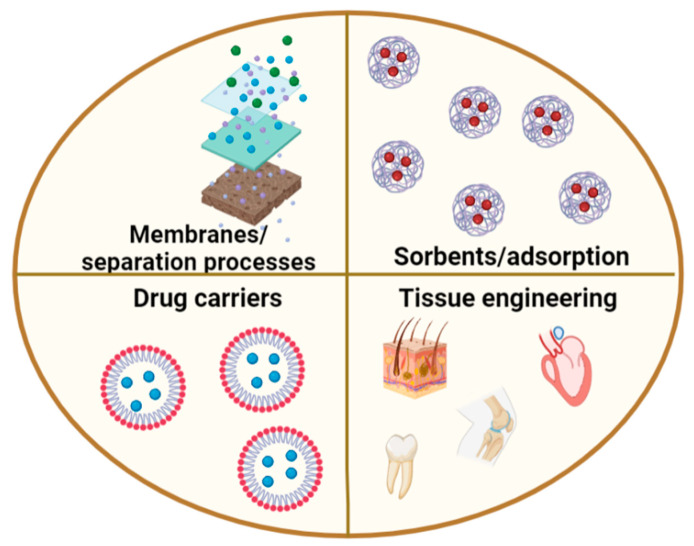
Applications for chitosan materials.

Due to the presence of reactive functionalities in the molecular chain, chitosan can be modified chemically or physically to produce scaffolds with desirable properties for tissue engineering. Chitosan-based scaffolds have been used to prepare high-resolution 3D patterns with adaptable pore sizes, interconnected lamellae and the ability to support the developmental lineage of encapsulated cells for use in the regeneration of bone, cartilage, vascular systems, neural networks and skin tissue [42,43,44]. The use of chitosan in medicine is supported by its various biological properties, including high biodegradability, nontoxicity, antifungal activity, accelerated wound healing capabilities, and stimulation of the immune system. Thus, chitosan has already been used in contact lenses, eye fluid, artificial skin, surgical sutures, artificial blood vessels, bandages, sponges, burn dressings, and hemostatic, antibacterial, and antifungal agents, as well as for blood cholesterol control, controlled drug release, accelerated wound healing, and tumor and plaque inhibition [42,43,44]. These applications for chitosan materials are summarized in Figure 3.

This review article summarizes the methods of preparation and application of pure porous chitosan materials. Increasing environmental awareness has highlighted the need to replace artificial polymer materials with natural polymers. Therefore, this review of chitosan-derived porous materials will support further research concerning these materials and the design of modified derivatives with unique applications. Although chitosan materials have been described in the literature, this review comprehensively describes the available methods for obtaining pure porous chitosan materials with specific structural features and their various applications. This article is divided into five sections according to the five strategies for preparing chitosan-based porous materials, i.e., cryogelation, freeze-drying, sol-gel, phase inversion, and extraction of a porogen agent. Each section contains a general description of the preparation method, as well as the characterization of the obtained chitosan structure. The potential applications and future prospects of specific materials are also discussed.

## 2. Freeze-Drying (Lyophilization)

Freeze-drying, also known as lyophilization, is a very common technique for preparing highly-porous materials with large specific surface areas [45,46,47,48,49,50,51,52,53]. This approach is relevant for obtaining materials with hierarchical structures because it enables control over the structure of the manufactured material [46,47]. Lyophilization is widely used in the pharmaceutical industry [48,49,50], the food industry [51,52], and biotechnology [53].

**Figure 4:**
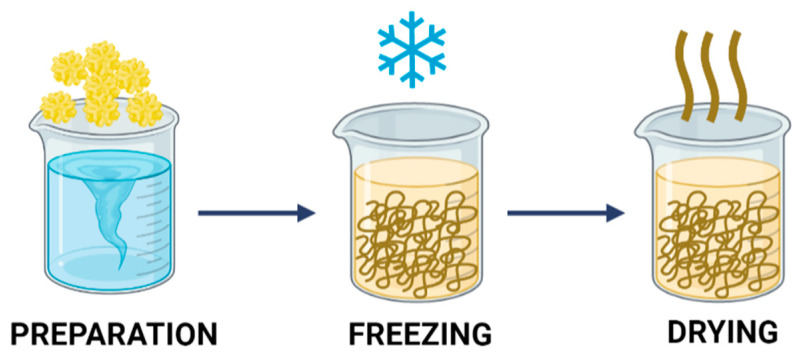
Formation of porous materials by lyophilization.

Lyophilization is based on the physical removal of the solvent from a frozen solution (or suspension) via sublimation. This results in a solid structure containing voids previously occupied by condensed solvent molecules. The preparation of porous materials using the freeze-drying method is presented in Figure 4.

The technique of preparation of porous materials by lyophilization begins with sample preparation, where the key parameters, i.e., silico precursor concentration, type of solvent and mixture ratio, affect the final structure of the material [54]. In the next steps, the sample, which is a solution or homogeneous suspension, is frozen [55] and dried to remove the condensed solvent [56]. This procedure yields a super-dried material that contains less than 0.5 wt.% water. 

### 2.1. Preparation of Chitosan-Derived Porous Materials by Lyophilization

The fabrication of porous chitosan materials via lyophilization involves three steps: preparation of chitosan solution, freezing, and removal of the frozen solvent by lyophilization. 

Depending on the operating parameters (i.e., initial chitosan concentration, freezing time, and freezing temperature), it is possible to produce a material with designed porosity and specific surface area. Garg et al. [57] employed lyophilization to prepare chitosan scaffolds. During the freeze-drying process, they consistently increased the operating temperature and decreased the pressure. They ultimately obtained porous chitosan scaffolds that were white in color (when dried), smooth, spongy, flexible, and sufficiently strong to withstand the drying process without undergoing deformation. In terms of the pore structure, the pores were heterogeneous and interconnected, and their diameters ranged from 10 to 20 μm. Porous chitosan scaffolds were also obtained by Nwe et al. [5] using various sources of chitosan, including shrimp shells, crab shells, squid bone plates, and mycelia. The solution was poured into a culture dish and frozen at a slow rate under relatively mild conditions. The samples were then lyophilized. To protect the chitosan scaffolds from dissolution in an aqueous medium, they were immersed in an alcoholic solution of NaOH. The results indicated that the diameter of all chitosan scaffolds after neutralization was approximately 16% smaller than that before neutralization. Scanning electron microscopy (SEM) images revealed heterogeneous 3D pore microstructures with well-connected pores in all chitosan scaffolds. Three types of pores were identified: perpendicular elongated pores, polygonal pores, and elongated pores. The perpendicular pores were produced from the highly parallel growth of ice crystals between the layers of the scaffold substrate, which resulted from the formation of hydrogen bonds between long polymer chains during the freeze-drying process. Polygonal pores and elongated pores occurred randomly in small numbers but influenced the reduction in scaffold thickness following neutralization. The substrate layers in all chitosan scaffolds adopted sheet-like structures. The average pore diameter of all chitosan scaffolds was in the range of 60–90 μm. Similarly, Song et al. [58] fabricated hydrogel porous chitosan beads and compared them to hydrogels prepared without freezing (i.e., direct lyophilization). In this case, freeze-drying generated large pores inside the chitosan microspheres and promoted the formation of a rough layer on their surface, whereas direct freeze-drying led to relatively small pores and a dense surface layer. These observations were explained based on the physical phenomena occurring during both processes. Freezing of the hydrogel involved a change in the state of water aggregation, which resulted in the formation of ice nuclei and, consequently, larger ice crystals. After sublimation, the large ice crystal volumes became large pores. In the case of direct lyophilization, the water inside the microspheres was distributed more evenly relative to the ice. Furthermore, the concentration of chitosan played an important role in governing the structure and morphology of the porous microspheres. A higher chitosan concentration led to a more compact structure and a smaller pore size, both on the surface and inside the chitosan microspheres. This phenomenon was explained by the balance between adsorption and desorption of solute molecules on the ice crystal surface, which significantly affected the growth of ice crystals. Meanwhile, as the concentration of chitosan increased, the tendency of chitosan to accumulate and adsorb on the ice crystal surface also increased, thereby inhibiting the growth of ice crystals. As a result, more ice crystals with smaller diameters were formed.

Tomaz et al. [59] applied a freeze-drying method to fabricate porous chitosan membranes for drug carrier applications. Specifically, a chitosan solution was poured into polystyrene Petri dishes, frozen for 24 h at −84 °C, and lyophilized for 72 h. The dry samples were removed from the Petri dishes and neutralized in NH_4_OH for 72 h. Finally, the samples were washed with distilled water. According to SEM images, the obtained membranes had a 3D interconnected macroporous structure. The mean pore size decreased as the degree of crosslinking in the membranes increased. A summary of the chitosan materials obtained via freeze-drying is presented in Table 1 with their structural characteristics and potential applications.

### 2.2. Application of Porous Chitosan Materials Prepared by the Freeze-Drying Method

In recent years, the lyophilization technique has been used to obtain chitosan-based porous materials that serve as scaffolds [63,64,65], microspheres [58,73,74], and membranes [59,60]. Tissue engineering is one of the most popular fields in which porous chitosan materials are applied [61,62,68]. The rich pore structures of the chitosan scaffolds provide an excellent environment for cell growth. Moreover, the uniform shape of the pores and high specific surface area facilitates the adhesion of cells on the internal walls of the scaffolds [75,76]. Scientists have also noticed a correlation between the elasticity and stiffness of the extracellular matrix (i.e., chitosan scaffold) and the quantity of adsorbed Schwann cells [77]. Furthermore, various chitosan properties, such as its biocompatibility, antibacterial activity, or ability to promote wound healing significantly impact the vitality and growth rates of cells. Thus, chitosan scaffolds obtained via freeze-drying offer the potential for culturing various types of cells [63,78,79]. 

Another important field of science where chitosan-based porous materials show wide applicability is the pharmaceutical industry, in particular, controlled drug release. Chen et al. [80] studied the compatibility between chitosan xerogel and doxorubicin hydrochloride (DOX), which is an anticancer drug, with the goal of developing a drug delivery system. They found that the chitosan xerogel absorbed DOX owing to the presence of –NH_2_, –OH, and phenolic groups. In addition, as the drug concentration increased, the chitosan material was able to store significantly more DOX. This study demonstrated that the DOX release rate decreased over time, and after 28 days, the cumulative drug release reached almost 50% at pH 7.4. The sorption capacity for DOX and the long-term release capacity of the chitosan xerogel were closely related to the structure and morphology of the carrier. As the specific surface area increased, the absorption capacity also increased, and consequently, the drug was released more slowly.

Porous chitosan materials obtained using lyophilization have also been used to remove ions from water. Ren et al. [74] studied the relationship between the structure of porous chitosan microspheres and their adsorption of Cr(VI) ions. They observed that the chitosan matrix’s adsorption affinity for Cr(VI) ions was related to the overall porosity of the beads. The microspheres with the highest porosity exhibited the highest adsorption capacity. This phenomenon was explained by the fact that beads with higher porosity contained more free –NH_2_ groups, which adsorbed Cr(VI) ions.

Zeng et al. [60] indicated the possibility of using porous chitosan membranes for heterogeneous catalysis. They confirmed that the obtained material was highly active, selective, and recyclable in the cross-coupling reactions of aromatic halides with alkenes. In this case, Pd(II) ions served as chitosan crosslinking agents and as catalytic sites for the cross-coupling reactions. The excellent activity and stability of the chitosan membranes were attributed to their highly-porous structures and the strong chelation between palladium and numerous hydroxyl and amine functional groups in the chitosan-based macromolecules.

## 3. Cryogelation

**Figure 5:**
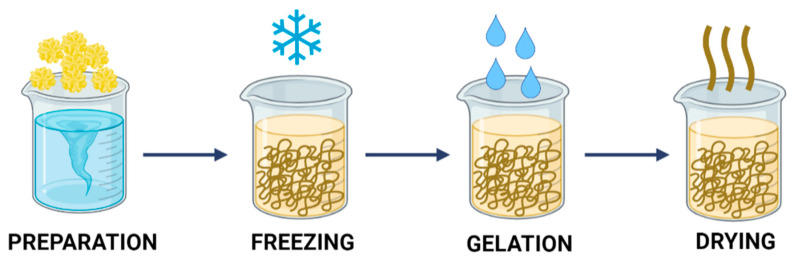
Formation of porous materials by cryogelation.

Another method that allows for the preparation of porous materials is cryogelation. This approach is attractive because of its inherent time and energy efficiency, as well as its scalability. In addition, this technique is simple and allows control over the pore structure depending on the cooling rate during the initial stages [81,82,83]. The cryogelation process involves the following steps: polymer solution preparation, freezing, gelation, and drying. Freezing represents a very important stage, which has a significant influence on the structure of the obtained material. The cooling rate determines the size of the ice nuclei and the corresponding number and diameter of the pores. The frozen sample is then immersed in a non-solvent solution to induce gelation, which is defined as the formation of intermolecular or intramolecular bonds. This process makes the polymer network more compact, inhibits dissolution, and reduces the swelling of the material [84,85]. Moreover, the gelation process protects the pore structure from collapse during the phase separation step [86]. The pores formed in the cryogelation process are typically large because of the increase in water volume during solidification. Nevertheless, their diameters can be controlled within a certain range by adjusting the cooling rate during the second stage (i.e., freezing). A scheme showing the process of fabricating porous materials by a cryogelation method is presented in Figure 5.

### 3.1. Preparation of Chitosan-Derived Porous Materials via Cryogelation

Ho et al. [86] prepared porous chitosan scaffolds using a cryogelation method, where the chitosan solution was frozen at −20 °C. To induce gelation below the melting point of the polymer solution, the frozen samples were immersed in a pre-cooled NaOH/ethanol solution. After removing the non-solvent, the chitosan scaffolds were dried completely at room temperature. The final structure contained interconnected pores with diameters in the range of 60–150 μm and a high porosity (~80%). The authors also noted the relatively high robustness of the porous structure of the material obtained after gelation. Chen et al. [87] studied the influence of the type of organic acid (as a solvent for chitosan) on the structure and properties of the resulting porous chitosan materials. They tested six carboxylic acids (acetic, ascorbic, citric, glycolic, malic, and tartaric acids) under identical conditions. In each case, the chitosan solution was frozen at −80 °C for 12 h. The frozen samples were then immersed in a NaOH/ethanol mixture at −20 °C for 12 h, and after crosslinking, they were washed in saline phosphate buffer. Porosity measurements indicated that the free volume inside the membranes was generally consistent, regardless of the acid used to prepare the solution, and in all cases, this parameter was slightly higher than 90%. In terms of the mechanical properties of the porous membranes, those derived from chitosan dissolved in ascorbic acid were the most durable (strength ≈ 8 N/g). The authors proposed that this was related to the oxidation of the two hydroxyl groups to carboxyl groups in the ascorbic acid environment; this oxidation also promoted the initial crosslinking of chitosan particles. Pinto et al. [88] studied porous chitosan scaffolds crosslinked using different concentrations of glutaraldehyde. In this case, the mixture of chitosan and glutaraldehyde was frozen at −20 °C for 24 h, transferred to a freeze-dryer, and stored at −52 °C for four days. The lyophilized samples were rehydrated and stabilized with ethanol, re-frozen in liquid nitrogen, lyophilized for an additional 24 h, and stored in a desiccator. The SEM microphotographs of the chitosan scaffolds revealed interconnected pores ranging in size from 50 to 200 μm. The addition of glutaraldehyde did not significantly alter the morphology or size of the pores, and therefore, clear interconnections between pores were maintained. Furthermore, the addition of glutaraldehyde increased the compressive strength of the scaffolds because glutaraldehyde reacted with chitosan, making the structure more rigid and mechanically resistant. Moreover, a gradual increase in Young’s modulus was observed as the crosslinking agent concentration increased.

### 3.2. Application of Porous Chitosan Materials Prepared by Cryogelation Method

Ho et al. [86] employed porous chitosan scaffolds obtained by a cryogelation method for cell culture applications. Cell culture experiments were conducted using ROS cells, which are osteoblast-like cells that can mineralize and/or express collagen and osteocalcin. Their results indicated that ROS cells cultured in chitosan-based scaffolds could attach, spread, and proliferate, thus demonstrating their potential applicability in tissue engineering. Pinto et al. [88] confirmed that porous chitosan scaffolds crosslinked with glutaraldehyde could be used in bone tissue engineering. The obtained scaffolds had sufficient biocompatible properties and enhanced biological performance. According to biological assessments, the material containing a low concentration of glutaraldehyde achieved the best biological performance in terms of metabolic activity, ALP expression, cell morphology, cell/scaffold interactions and gene expression.

## 4. Sol-Gel Method 

**Figure 6:**
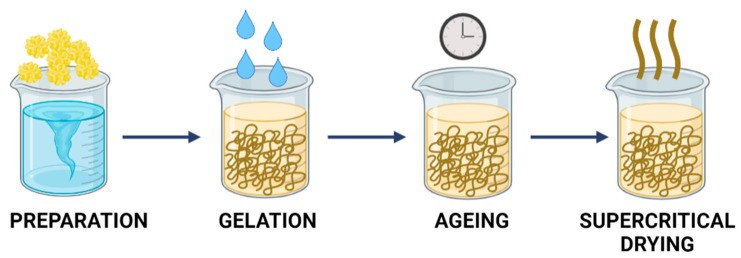
Formation of porous materials by the sol-gel method.

The sol-gel method enables powder-free processing of glasses, ceramics, thin films, or fibers directly from a solution. The precursors are mixed at the molecular level, and different material shapes can be formed at much lower temperatures than what is possible with traditional methods [89,90,91]. The specific steps of this method are similar to those in the freeze-drying and cryogelation approaches, except that the solution is not frozen, and the phase separation stage is significantly different. Specifically, a gel is dried under supercritical conditions for the solvent, which leads to the formation of a porous structure (i.e., aerogel). Sol-gel processing begins with the preparation of a solution or sol that later becomes a gel. The solution may comprise inorganic salts or organic compounds, which are then hydrolyzed and condensed to form a sol or gel. The gel state indicates a three-dimensional interconnected solid network where liquid fills the pores. In the wet gel state, these pores are interconnected. Aerogels represent a prominent group of porous materials [92]; they are unique nanoporous substances with particular structures and special properties. When manufacturing such materials, the liquid portion of the gel is replaced by gas using an appropriate drying technology. The resulting solid material is ultralight, porous, and in some cases, transparent because it is approximately 99.2% empty space. A scheme illustrating the preparation of porous materials using the sol-gel method is shown in Figure 6.

### 4.1. Preparation of Chitosan-Derived Porous Materials Using the Sol-Gel Method

To produce chitosan aerogel, a colloidal solution (sol) is first prepared. Dilute solutions of simple organic acids represent solvents that are suitable for dissolving chitosan. The initial concentration of chitosan and the pH both significantly influence the final structure of the aerogel. In the next step, the solution undergoes gelation. The chitosan gelation is induced by changing the pH of the solution (physical gelation) or by adding various crosslinking agents (chemical gelation). Increasing the pH of the solution causes the detachment of protons from the –NH_3_^+^ groups of chitosan; as a result, the polymer does not dissolve upon contact with water and the sol transforms into a gel. In practice, the physical gelation of a chitosan solution is promoted by placing the chitosan solution under an ammonium atmosphere [93] or in a NaOH bath [94] for a certain amount of time to allow for solution condensation. Physical gelation can also be carried out under the influence of di-, tri-, or five-valent ions. For example, sulfuric acid(VI) induces electrostatic interactions between SO_4_^2-^ anions and positively charged amine groups –NH_3_^+^. This increases the tensile strength of the material without significantly reducing its hydrophilicity [95]. Other compounds that can promote ionic gelation of chitosan include sodium tripolyphosphate [96], citric acid [97], phosphoric acid, and oxalic acid [98]. Chemical crosslinking leads to the formation of new bonds between hydroxyl and amino groups in chitosan and corresponding functional groups in the crosslinker. These bonds create a tighter polymer network. The most common organic compounds used for the chemical gelation of chitosan include glutaraldehyde [99], formaldehyde [100], epichlorohydrin [101], genipin [102], and glyoxal [103]. 

The next step in the sol-gel process is gel aging. To further grow the structure, the partially gelled solution is left untouched for a period of a few hours to a few weeks, depending on the gelation conditions. During this time, condensation reactions proceed, and the partially gelled solution becomes increasingly viscous. To stabilize the porous gel structure and prevent the collapse of the pore walls during drying, solvent exchange must occur at an appropriate point during the aging process. In some cases, dilute aqueous acetic acid solution is exchanged for ethanol. Because of its lower surface tension and higher volatility relative to water, ethanol is more rapidly removed during drying. In the case of chemical crosslinking, the aging process occurs relatively quickly owing to the fast rate of reaction between functional groups in the chitosan and in the crosslinker. After complete condensation, the gel contains solvent molecules inside the structure, which are in thermodynamic equilibrium with the polymer. In the final step, the gel is dried. One of the most common techniques for drying hydrophilic gels is supercritical CO_2_ (scCO_2_) extraction [92,104,105]. 

A comparison of the structural properties and applications of chitosan aerogels is presented in Table 2.

López-Iglesias et al. [106] studied a series of chitosan aerogels using the sol-gel process with scCO_2_ extraction as the method of drying. The samples were prepared from a solution of chitosan in dilute acetic acid, which was then dropped into a 0.1 M NaOH solution (gelling bath). After a short gelation time, the hydrogel beads were transferred to an absolute ethanol bath for 30 min. The solvent exchange step was repeated to completely remove water from the alcogel beads. Finally, the chitosan beads were dried via scCO_2_ extraction, which afforded the final aerogels. The authors observed that the beads were formed based on a network of interpenetrating bundles of polysaccharide fibers. The dual porous structure of these aerogels was observed in the inner and outer structures of the particles, where the voids within and between the fiber bundles were mesoporous (2–50 nm) and macroporous (>50 nm), respectively. Moreover, the porosity value remained constant at approximately 97%, while the specific surface area (determined by the BET method) increased from 257 to 479 m^2^/g as the gel aging time increased. Takeshita et al. [112] prepared chitosan aerogels using mixed (physico-chemical) gelation in the presence of various amounts of urea as a coagulant agent. To initiate the thermal decomposition of urea and induce gelation, the prepared solution was sealed in an airtight polypropylene container and stored at 80 °C for 72 h. The resulting hydrogel was soaked in a water/ethanol mixture three times, with two exchanges per day. Then, the gel was placed in an autoclave and encapsulated in a scCO_2_ drying system. The SEM images of the aerogels revealed that samples with low urea concentrations exhibited mesoporous structures consisting of primary particles that were approximately 10 nm in diameter. This structure was highly homogeneous below the sub-micrometer scale. Aerogels with high urea concentrations consisted of similar primary nanoparticles, although they also formed fibrous and aggregate structures exceeding 100 nm in diameter, which were composed of primary nanoparticles. The BET surface area reached a maximum of 436 m^2^/g for the sample with the lowest urea concentration, and this area decreased (to ~300 m^2^/g) as the initial urea concentration increased. Another method for preparing porous chitosan aerogels by the sol-gel method is via hydrophobic modification [111]. The key point of this approach is the combination of the nanofibrous material and a hydrophobization agent, i.e., chitosan and alkyl aldehydes. In this process, a certain amount of alkyl aldehyde was mixed with methanol and then with an aqueous solution of formaldehyde and chitosan. The mixture was left overnight at 60 °C in a tightly sealed container. After cooling to room temperature, the resulting gel was washed by soaking in methanol with continuous stirring to remove water, and then dried supercritically in a CO_2_-methanol system at 80 °C and 20 MPa to obtain an aldehyde-modified chitosan aerogel. The SEM observations revealed entangled chitosan nanofibers (less than 50 nm in diameter) forming a mesoporous structure. The specific surface areas were 672 and 581 m^2^/g for the unmodified sample and the hexanal-modified sample, respectively. Obaidat et al. [110] used supercritical fluid technology to prepare porous chitosan aerogels. In this case, raw chitosan was dissolved in a dilute acetic acid solution, while chitosan oligomers were dissolved in water. The resulting solution was added dropwise to a sodium tripolyphosphate solution. The obtained chitosan microparticles were dehydrated by immersion in a series of ethanol baths with increasing alcohol concentrations. The microparticles were then dried with supercritical fluid at 9.997 MPa and 40 °C for 2 h. The SEM images showed that the obtained aerogels had a homogeneous shape with a high specific surface area thanks to macro- and mesopores. The specific surface area and porosity of raw chitosan were higher than those of the oligomers. It was therefore demonstrated that supercritical fluid drying overcame the limitations of traditional drying by maintaining high open porosity and favorable textural properties in the prepared porous structures, i.e., aerogels. This outcome was attained because a moderate temperature was used during drying, resulting in conformational changes and minimizing intermolecular interactions.

Another interesting way to prepare porous chitosan aerogels using the sol-gel method is to obtain them directly, without introducing catalysts or oxidants [108]. In this case, chitosan powder is first dissolved in acetic acid to form a homogeneous solution at the molecular level and then regenerated to form a gel through crosslinking. Formaldehyde was used as a crosslinker because it reacts with NH_2_ groups to form Schiff base moieties. The SEM images showed that the obtained chitosan aerogels comprised entangled, i.e., randomly-oriented, nanofibers that were 5–10 nm in diameter and mesopores that were 10–50 nm in size. The pore area increased with decreasing apparent density, ultimately reaching 545 m^2^/g. X-ray diffraction profiles showed that the chitosan nanofibers in the aerogels were almost amorphous.

### 4.2. Application of Porous Chitosan Materials Prepared by Sol-Gel Method

Chitosan-based aerogels represent very promising materials with high specific surface area, porosity, and biocompatibility. As a result, they have potential applicability in various fields of science and contemporary technologies [106,107,108,109,110,111]. The main advantages of using chitosan for aerogel preparation are (i) facile control over gelation through suitable pH selection, (ii) abundance of hydroxyl and amino groups, (iii) simplicity of chemical modification, and (iv) wide selection of potential crosslinking agents. López-Iglesias et al. [106] obtained porous chitosan aerogels for chronic wound applications. They noted that vancomycin-containing chitosan aerogels comprise a promising formulation that can be incorporated into dressings for treating chronic wounds, especially when infectious episodes are expected. Importantly, loading vancomycin into chitosan aerogels did not affect vancomycin colonization at the wound site. Furthermore, this approach can provide rapid local delivery of the antibiotic at the wound site to prevent infection soon after wound debridement, without compromising cell viability. Obaidat et al. [110] confirmed that supercritical fluid technology could be employed to produce chitosan aerogel microparticles with salbutamol, which may be useful in pulmonary drug delivery systems. They observed prolonged release in all prepared microparticles compared with unformulated salbutamol. The release profile of salbutamol from the developed material was influenced by the type of chitosan and the sodium tripolyphosphate concentration. The highest release rate was obtained for raw chitosan because it had the highest porosity. Increasing the sodium tripolyphosphate concentration decreased the salbutamol release rate thanks to the increased crosslinking of the polymer chain, which reduced the swelling capacity. Zhang et al. [107] fabricated chitosan aerogels for the adsorption of methyl orange from water tests. Their investigations revealed that the prepared adsorbents had excellent adsorption properties because of their high specific surface areas. The main driving forces for methyl orange adsorption were physical or hydrogen bonding interactions and the high specific surface area, which contributed to favorable three-dimensional structures that offered numerous adsorption sites.

## 5. Phase Inversion

**Figure 7:**
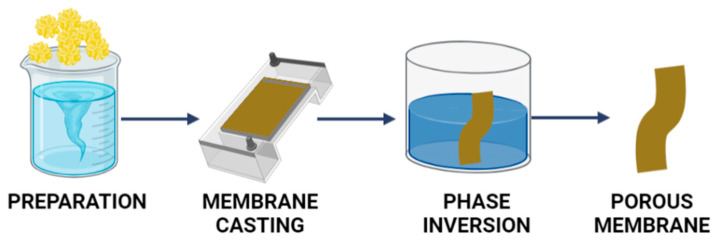
Formation of porous materials by phase inversion.

The wet phase-inversion method is the most widely used technique for preparing porous materials. Four strategies can be applied for the formation of phase-inversion membranes: immersion precipitation, vapor-phase precipitation, precipitation by controlled evaporation, and thermally-induced phase separation [113,114,115]. Immersion precipitation is the most commonly used approach to polymeric membrane preparation. In this method, the polymer solution is cast onto a suitable support layer and then immersed in a coagulation bath containing a non-solvent. Precipitation occurs by exchanging the solvent with the non-solvent. In vapor-phase precipitation, the main phenomenon responsible for phase separation is the influx of non-solvent to the membrane. The non-solvent penetrates from the vapor phase as water, acetone, or ammonia vapor. In this case, the system enters a diphasic regime due to non-solvent intake, rather than solvent loss. Precipitation by controlled evaporation can be implemented to prepare materials with less porous surfaces. The evaporation step forms a “skin layer” with locally increased polymer concentrations following the selective loss of the volatile (co)solvent. After immersion in the coagulation bath, the skin layer forms a resistance barrier between the main part of the membrane and the coagulation bath. Thus, non-solvent and solvent diffusion are hindered and demixation is delayed. In thermally-induced phase separation, polymer precipitation is induced by a reduction in temperature, which is usually initiated by immersing the material in a quenching bath. To prepare membranes, it is necessary to find a substance that cannot dissolve the polymer at room temperature but does behave as a solvent close to the melting point of the polymer. Reducing the temperature therefore triggers phase inversion. The general procedure for fabricating porous materials by phase inversion is presented schematically in Figure 7. 

### 5.1. Preparation of Chitosan-Derived Porous Materials Using Phase Inversion

Mi et al. [116] prepared porous chitosan membranes via dry/wet phase separation. For dry phase separation, the casted chitosan solution was pre-heated in an oven with circulating hot air (50 °C) for 10–60 min. The obtained material was immersed in an aqueous NaOH (2 wt.%)-Na_2_CO_3_ (0.05 wt.%) solution, which was used as a coagulant for 24 h. After coagulation, the chitosan membrane was soaked in distilled water to remove NaOH, and then it was freeze-dried. The obtained membranes had asymmetric structures; a dense skin was formed during dry inversion, and a sponge-like porous layer formed following wet phase separation. The thickness of the skin and sponge-like porous layers depended on the evaporation time. Specifically, a longer “dry” evaporation time increased the thickness of the skin layer while simultaneously decreasing the thickness of the porous layer. Asymmetric gradational-changed porous chitosan materials were described by Hong et al. [117]. In this case, a chitosan solution was cast into a glass plate and pre-vaporized in an oven at 50 °C for 1 h. The obtained membrane was immersed in a NaOH solution for 24 h, then washed repeatedly with deionized water, and freeze-dried for 2 h. The SEM analysis showed that the studied membranes contained three layers: a dense layer (~25 μm), a transitional medium-density layer (~30 μm), and a spongy porous layer (~100 μm). The medium-density transition layer contained numerous interconnected micropores, approximately 1–2 μm in diameter, whereas the interconnected pores in the porous spongy layer were approximately 15–60 μm in diameter. Porous chitosan membranes can also be prepared via cryogenic-induced phase separation [118]. In this case, the mixture of chitosan and glutaraldehyde was cast onto a glass plate and placed in a chamber at low temperature for 72 h until crosslinking was complete. The obtained membrane was immersed in acetone three times to extract ice and decompressed at room temperature to remove acetone. Finally, it was neutralized with NaOH solution to remove any remaining acetic acid, washed with deionized water until pH 7, and dried. The SEM images revealed a 3D porous structure, stacked by the porous chitosan lamellas. The thickness of the prepared membranes was 80 μm, and the cross-sections indicate long narrow pores oriented parallel to the membrane surface. The surface exhibited a homogeneous porous structure, which was similar to a lacy structure. The porosity was 74.2% and originated from an open-pore membrane structure with high porosity. 

The thermally-induced phase separation method for fabricating porous chitosan membranes was studied by Qin et al. [119]. A chitosan solution was poured into a Petri dish and immediately quenched to −10 to −20 °C for 6–12 h. At −20 °C, the chitosan/water-ethanol solution became a relatively transparent soft gel. To regenerate the chitosan, a pre-cooled (0 °C) coagulant solution was added, and the mixture was maintained at 0 °C for 6 h. The gel membrane was subsequently frozen at −20 °C for 2 h, and then lyophilized at −50 °C for 24 h. The SEM images indicated that because of the phase separation into two bicontinuous polymer-rich (chitosan) and solvent-rich (water) phases at low temperature (−20 °C), chitosan formed a skeleton of pores, and water solidified into ice crystals within the chitosan matrix. As a result, large holes existed in the chitosan material. The pore sizes could be modulated by replacing water with a mixture of water and ethanol. This phenomenon was a result of rapid ice crystallization, which led to a chitosan film with a large number of pores. 

### 5.2. Application of Porous Chitosan Materials Prepared by a Phase Separation Method

Hong et al. [117] studied porous chitosan membranes for guided periodontal tissue regeneration. The investigated membranes had excellent surface and structural compatibility. Moreover, they did not cause anaphylactic reactions or hemolysis and exhibited no cytotoxicity or pyrogenic effects. Furthermore, their bioactivity induced and guided tissue regeneration when combined with growth factors and other bioactive elements. In terms of membrane degradation, the obtained materials maintained their structural integrity for 5–6 weeks in an RLS enzyme solution, which satisfied the requirements for a guided tissue regeneration membrane for curing periodontal disease. The other application of porous chitosan materials prepared by phase inversion is controlling the antigen release of the Newcastle disease (ND) vaccine [120]. Indeed, controlled-release of the ND vaccine antigen could be achieved by adsorbing the antigen onto chemically-modified porous chitosan microspheres. These results indicated that either quaternary or succinyl porous chitosan microspheres could form strong electrostatic attractions or intermolecular interactions with the WP virus antigen, thereby enabling a very slow rate of antigen release. Moreover, the rate of antigen release from the microspheres was not significantly dependent on enzymatic degradation. Salerno et al. [121] investigated porous chitosan membranes in the context of human epidermal stratification and differentiation applications. They demonstrated that it was possible to stimulate the formation of epidermal layers by using membranes with tailored properties. Distinct effects on cell proliferation and differentiation of human keratinocytes could be observed depending on the surface topography of the nano- and microstructured membranes. Nanoporous membranes with pore diameters of 26 nm exhibited moderate roughness and elastic moduli, which allowed them to influence the development of a cornified epidermal surface layer structure characterized by low thickness, low cell proliferation, and high CK1 expression. In contrast, microporous chitosan membranes with open-pore diameters of 0.131 µm and increased roughness and elastic moduli induced the formation of basal epidermal lamina, with numerous proliferating cells that stratified and differentiated over time, thus forming a multilayered and viable epidermis. 

The dry/wet phase-inversion method was also applied to generate asymmetric porous chitosan membranes for controlled antibacterial release [117]. The results indicated that a silver sulfadiazine (AgSD)-doped asymmetric chitosan membrane represents a potential wound dressing material with antimicrobial properties to protect injured skin from infection. This membrane allowed the burst-release of sulfadiazine and the slow release of silver from the membrane into the burn wound to control bacterial growth. In addition, it was less cytotoxic than the traditional AgSD cream and more effective in terms of long-term growth inhibition of *Pseudomonas aeruginosa* and *Staphylococcus aureus* infections in the wound. Thermal phase separation was also used to fabricate porous chitosan materials to study their adsorption behavior for Cu^2+^ and their potential for soft tissue engineering applications [119,122]. It was confirmed that porous chitosan membranes are promising adsorbents for treating wastewater contaminated with heavy metal ions. These membranes exhibited high adsorption capacity for Cu^2+^, and the adsorption process was based on monolayer and chemical adsorption. The chitosan-based materials also promoted fibroblast proliferation and were therefore considered promising scaffolds for soft tissue engineering. The mechanical environment of the scaffolds was intentionally soft so that the cells could not generate large traction forces when they attached to and pulled on the surface. This resulted in a more rounded cell morphology, which better mimics the native morphology of fibroblast cells in soft human tissue.

## 6. Extraction of a Porogen Agent 

**Figure 8:**
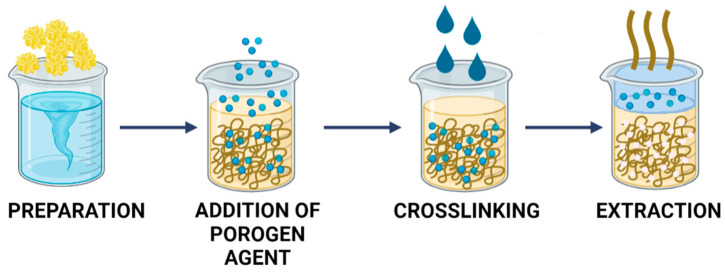
Formation of porous materials by adding a porogen agent.

Another interesting way to prepare porous membranes is by adding a porogen agent to the polymer solution. Introducing a porogen agent as a porosity former is a facile, convenient, and cost-effective method that can be applied with a wide range of polymers. Furthermore, porogen agents determine the stability, selectivity, and permeability of the polymer. A porogen is an organic or inorganic material that is added to the polymer solution before membrane formation. During membrane shaping, it is selectively removed by leaching, thereby forming pores in the place of porogen molecules in the membrane matrix. In this process, the pore size is controlled by the size of the porogen. Some of the most popular porogen agents are NaCl, sugar crystals, paraffin spheres, and polymers. The most widely used porogen is NaCl, although it is not stable enough to obtain uniformly-sized pores, and therefore, its applicability in tissue engineering is limited. For this reason, new porogen agents are still being developed and tested to obtain membranes with uniform pore sizes [123,124,125,126]. The scheme in Figure 8 illustrates the preparation of porous materials by the addition of a porogen agent.

### 6.1. Preparation and Characterization of Chitosan-Derived Porous Materials Prepared by the Extraction of a Porogen Agent

Porogen agents have also been used to fabricate porous chitosan membranes. Chao et al. [127] added NaCl particles to a 5% *v*/*v* chitosan solution. The obtained membranes were then dried at 50 °C, immersed in an aqueous 1 M NaOH solution, and then immersed in deionized water to dissolve the NaCl particles and remove the remaining NaOH. As a result, chitosan membranes with macroporous structures were obtained. In this case, the addition of NaCl particles to the chitosan solution led to a more compact polymer conformation and reduced the solubility of the polymer chains. Furthermore, some of the NaCl particles dissolved in the chitosan solution to form amorphous polymer precipitates, whereas others helped form the macroporous structure. Overall, the number of NaCl particles had a significant effect on the porosity of the resulting chitosan membranes. The mean pore diameter of the investigated membranes ranged from 74.14 to 153.38 μm, and the pore size generally depended on the physico-chemical treatment applied during membrane preparation (oven, freeze, and vacuum dry steps). Another porogen agent that is commonly used to obtain macroporous chitosan membranes is silica [128]. Silica particles could be removed from the dense membranes following treatment with a 5 wt.% solution of NaOH at 60 °C for 4 h. Next, the obtained membranes were washed with distilled water at 60 °C and again at room temperature. To properly plasticize the membranes to avoid pore closure during the drying process, the membrane materials were immersed in 20 wt.% glycerol solution for 30 min. The addition of silica particles to the polymer matrix induced the formation of macroporous chitosan membranes with asymmetric structures. The top layer was dense and non-porous, whereas a porous layer was formed at the bottom surface because silicon particles tended to fall within the chitosan film. This membrane structure served to increase the mechanical resistance and selectivity of the membrane. According to SEM observations, the pore size was controlled within the range from 4 to 20 μm based on the silica particle diameter. 

Zeng et al. [129] prepared porous chitosan membranes using polyethylene glycol as a porogen agent. They dissolved chitosan in 2 wt.% acetic acid and mixed this solution with various amounts of polyethylene glycol. To form the desired porous structure, the polyethylene glycol was removed by immersing the obtained membranes in a water bath at 80 °C for 8 h. The SEM images showed that the membrane surface adopted a 3D porous structure comprising stacked porous lamella. The ultimate pore size depended on the molecular weight of the polyethylene glycol. In general, the higher the molecular weight of polyethylene glycol, the larger the final pore size, thus indicating poorer compatibility with chitosan. An alternative porogen agent for preparing porous chitosan materials is sodium acetate [130]. In this case, the chitosan solution was mixed with sodium acetate salt, frozen at −70 °C for 24 h, and lyophilized for an additional 24 h. The chitosan-salt mixture was washed in ethanol for 2 h and then underwent salt-leaching in distilled water for 48 h. The amount of sodium acetate had a significant impact on the structure, porosity, and mechanical properties of the obtained porous chitosan material. The porosity increased as the sodium acetate content increased; material prepared with 90% sodium acetate contained larger primary pores (200–500 μm), as well as numerous smaller pores (7–30 μm). Compared with the phase-separation method, the use of sodium acetate as a porogen agent to prepare chitosan materials could increase the elongation-at-break and elastic recovery of the final material. Furthermore, more favorable mechanical properties were observed when using larger amounts of sodium acetate. 

Liu et al. [131] used eggshell membrane powder (ESMP) as a porogen agent to fabricate porous chitosan materials. Importantly, ESMP is a natural material that has attracted increased attention in many contexts owing to its nontoxicity, biocompatibility, and abundant functional groups. It can serve as a suitable porogen agent because it partially dissolves in NaOH solution. For example, chitosan solution (3.5% *w*/*v*) was mixed with an aqueous ESMP solution (7% *w*/*v*) and stirred for 2 h. Then, the mixture was treated with 10% NaOH solution, and the obtained mesoporous material was sequentially washed with deionized water, ethyl alcohol (50% *v*/*v*), and deionized water to reach neutrality. After adding ESMP to the chitosan matrix, the density of macropores increased, and the size range of macropores became narrower. Furthermore, ESMP promoted the transformation of macropore walls and dense lamella to a porous network structure with thinner gauze-like nets at the boundaries of the ESMP particles. The obtained structure was beneficial in terms of the diffusion of substrate molecules from the solution into the interior of the macroparticles. Additionally, ESMP loading on the surface gradually enhanced the roughness of the surface morphology. Finally, BET analysis showed that the changes to the mesopore structure of the chitosan material after adding ESMP were due to the dissolution of ESMP in the NaOH solution; when these particles were removed, larger spaces were created. On the other hand, ESMP induced attraction effects between chitosan chains, which reduced mesopore formation following the accumulation of chitosan chains. 

### 6.2. Application of Porous Chitosan Materials Prepared by Extracting a Porogen Agent

Recently [127], researchers demonstrated the higher adsorbing capabilities of porous chitosan materials prepared using NaCl particles as the porogen agent, relative to copper (II) ions. They noticed that Cu^2+^ ions sorbed on the surfaces of existing pores and between the segments of the matrix. This contributed to a significant improvement in the sorption capacity of the developed porous chitosan material. Shi et al. [132] observed that chitosan materials with a sponge-like 3D pore structure exhibited better sorption capacity for dyes. The porous chitosan-based membranes, which were prepared using polyethylene glycol as the porogen agent, indicated the possibility of combining high adsorption capacity with high-speed dynamic dye removal. This phenomenon was mainly attributed to the rejection of the millipores and the interactions between certain characteristic functional groups in the membrane and dye. The prepared membrane was also capable of repeated dye removal (>80% after 5 cycles of dynamic adsorption at a dye concentration of 150 mg/L). The same porogen agent (polyethylene glycol) was used to prepare porous chitosan membranes for filtration applications [133]. The incorporation of polyethylene glycol increased the hydrophilicity of the membranes, while the accumulation of polyethylene glycol molecules at the membrane surface stabilized the surface and generated a hydrophilic surface network, which increased pure water permeability. More favorable results were obtained when using a low concentration of polyethylene glycol because this enabled the creation of a membrane structure with a uniform morphology (no defects). 

Macroporous chitosan membranes prepared using a silica porogen agent have been applied in the context of drug permeation [128]. Observed differences in drug permeability were generally due to their differing water solubilities. Because the permeation process is governed by the distribution of the solvent in the water-filled pores, a more water-soluble drug will permeate more easily. Additionally, the permeation increased as the temperature increased because of the enhanced thermal mobility of the polymer, which promoted mass transport through the membrane. An analogous porous chitosan-silica membrane was investigated for the fabrication of batteries [134]. The results of this study indicated that a porous chitosan membrane prepared by silica removal could be implemented to construct coin cell proton batteries with excellent electrochemical properties and overall battery performances. In addition, the specific discharge capacities of the fabricated proton battery increased because of the absorption of H^+^ ions in the porous chitosan membrane at the electrode-membrane interface. This behavior was possible thanks to the relatively large pore size, high water absorption, high conductivity, and optimal linear sweep voltammetry of the novel material.

## 7. Summary

Porous chitosan materials, which are characterized by biocompatibility, nontoxicity, antibacterial properties, and biodegradability, are increasingly being considered for broad industrial applications. This review provides in-depth overviews of the developed porous chitosan materials while focusing on the methods of preparation, characterization, and application of these materials. The scope was limited to pure chitosan materials where the crosslinking agent was the only modification. This assumption was implemented to clearly discuss the direct links between the properties of the obtained material and the method (conditions) of preparation, without the influence of additional factors. In the descriptions of each method, we elucidate how the fabrication approach impacts the structure and potential applicability of porous chitosan materials. Overall, five membrane preparation methods were presented: cryogelation, freeze-drying, sol-gel, phase inversion, and extraction of a porogen agent. The basic principles of each process and the resulting porous chitosan materials were discussed in terms of their structures and applications. 

The information compiled in this article can be used as a guide to improve the structural and mechanical properties of chitosan materials to broaden the scope of their potential applications to other areas of industry.

## Figures and Tables

**Table 1:** Structure and applications of chitosan-derived porous materials obtained by freeze-drying.

Type of Material	Pore Characteristics	Porosity (%)/Pore Size (μm)	Application	Reference(s)
Membranes	Open-pore structure; oblong pores; slice building of the membrane	70–80/-	Catalysis (catalyst site for Heck coupling reaction)	[60]
Membranes	Interconnected macropores; uniform pore structure; fewer pores on the surface of the membrane	-/-	Drug release, inhibition of cancer cell growth	[59]
Scaffolds	Relatively small, interconnected pores; sponge-like scaffold structure	-/10–20	Drug delivery, tissue engineering	[57]
Scaffolds	Polygonal and interconnected pores; sheet-like scaffold structure; randomly-located pores	88–97/60–90	Biomedicine, cell proliferation	[5]
Scaffolds	Uniform, spherical pores; open-pore structure; pore size depended on initial chitosan concentration	-/40–250	Tissue engineering	[61]
Scaffolds	Small oblong pores that were heterogeneously distributed; specific surface area calculated using BHJ desorption isotherms (2.19 m^2^/g)	-/-	Bioengineering	[62]
Scaffolds	Medium-sized pores; uniform sponge-like scaffold structure	-/50–200	Tissue engineering (nerve regeneration)	[63]
Scaffolds	Interconnected pores with thin walls; pores on the scaffold surface and inside the structure	75–85/60–80	Cell proliferation, tissue engineering	[64]
Scaffolds	Spherical, interconnected pores; uniform scaffold structure; narrow range of pore sizes	30–80/105–138	Tissue engineering	[65]
Scaffolds	Interconnected pores; organized structure throughout material	-/-	Protein coating, drug delivery	[66]
Scaffolds	Interconnected pores; highly-porous structure; wide distribution of pore sizes	85–95/50–150	Cell proliferation	[67]
Scaffolds	Interconnected pores; sponge-like scaffold structure	-/100–200	Tissue engineering (bone regeneration)	[68]
Scaffolds	Structure with open, interconnected pores; round, uniformly-shaped pores	-/100–200	Cell proliferation (chondrocytes growth)	[69]
Scaffolds (dried hydrogels)	Uniform, homogenous pores with thin walls; pore size depended on the lyophilization conditions	-/9–400	-	[70]
Particles	Small, randomly dispersed pores within particles; pores on the particle surface were larger than interior pores	-/1.5–15	Cell proliferation	[71]
Nanofibers	Irregularly-shaped pores crucial for the structure; pore volume between 13.89–33.32 mL/g depending on initial chitosan concentration	-/20–180	Ion adsorption (copper ion removal)	[72]
Microspheres (microcarriers)	Pores distributed evenly on the surface and within microspheres; pores collapsed with higher initial concentrations of chitosan	90/15–20	Cell proliferation (hepatocyte growth)	[73]
Microspheres	Shape and structure of pores depended on chitosan concentration; uniformly distributed pores with spherical or oblong shapes	23.14–63.15/-	Ion adsorption (hexavalent chromium removal)	[58]
Microspheres	Shape and structure of pores depended on chitosan concentration, freezing temperature, and time; sponge-like interior structure and porosity >90% for 1 wt.% chitosan; uniform distribution of pores and porosity ~50% for 2.0–3.5 wt.% chitosan	45–95/-	Ion adsorption (hexavalent chromium removal)	[74]

**Table 2:** Structural properties and application of chitosan aerogels prepared using scCO_2_ extraction.

Gelation	Structural Characteristics	Porosity (%)/Pore Size (nm)	Application	Reference(s)
Aqueous solution of NaOH	Structure comprising numerous fibers with meso- and macropores; fibers distributed uniformly generating a structure with high specific surface area (257–479 m^2^/g) and specific pore volume (1.01–1.70 cm^3^/g)	96.7–97.2/ 2–50 and >50	Chronic wound healing	[106]
Glutaraldehyde	Chitosan aerogel comprising entangled nanofibers with diameters ~40 nm; interconnected fibers create structure with large specific surface (658–973 m^2^/g)	-/ 50–120	Adsorption of methyl orange	[107]
Glutaraldehyde	Chitosan aerogel structure comprising short nanofibers with diameters 5–10 nm; high specific surface area (545 m^2^/g) and thermal conductivity coefficient (0.022 W/mK)	86–97/ 10–50	House windows, glass walls of buildings, car windows	[108]
Formaldehyde	Aerogel structure comprising nanofibers with diameters ~15 nm and nanoparticulate aggregates; highly transparent, yellow aerogel with high specific surface area (737–872 m^2^/g)	89.3–96.0/ 21.3–43.6	Sound absorption	[109]
Sodium tripolyphosphate	Low-porosity aerogels with low specific surface area (73–103 m^2^/g); uniform aerogel structure with homogeneous pore shapes	20–29/-	Drug delivery	[110]
Formaldehyde and alkyl aldehyde	Aerogel with uniform, interconnected pores; structure comprising nanofibers with diameters ~50 nm; high specific surface area (581–672 m^2^/g)	-/-	Thermal insulation	[111]

## References

[1] Kou S., Peters L., Mucalo M. (2022). Chitosan: A review of molecular structure, bioactivities and interactions with the human body and micro-organisms. Carbohydr. Polym..

[2] Kou S., Peters L.M., Mucalo M.R. (2021). Chitosan: A review of sources and preparation methods. Int. J. Biol. Macromol..

[3] Boudouaia N., Bengharez Z., Jellali S. (2019). Preparation and characterization of chitosan extracted from shrimp shells waste and chitosan film: Application for Eriochrome black T removal from aqueous solutions. Appl. Water Sci..

[4] Huq T., Khan A., Brown D., Dhayagude N., He Z., Ni Y. (2022). Sources, production and commercial applications of fungal chitosan: A review. J. Bioresour. Bioprod..

[5] Crognale S., Russo C., Petruccioli M., D’Annibale A. (2022). Chitosan Production by Fungi: Current State of Knowledge, Future Opportunities and Constraints. Fermentation.

[6] Elsoud M.M.A., Kady E.M.E. (2019). Current trends in fungal biosynthesis of chitin and chitosan. Bull. Natl. Res. Cent..

[7] Hahn T., Tafi E., Paul A., Salvia R., Falabella P., Zibek S. (2020). Current state of chitin purification and chitosan production from insects. J. Chem. Technol. Biotechnol..

[8] Rasti H., Parivar K., Baharara J., Iranshahi M., Namvar F. (2017). Chitin from the Mollusc Chiton: Extraction, Characterization and Chitosan Preparation. Iran J. Pharm. Res..

[9] Kim J.S., Lee S. (2019). Immobilization of Trypsin from Porcine Pancreas onto Chitosan Nonwoven by Covalent Bonding. Polymers.

[10] Chang X.X., Mubarak N.M., Mazari S.A., Jatoi A.S., Ahmad A., Khalid M., Walvekar R., Abdullah E.C., Karri R.R., Siddiqui M.T.H. (2021). A review on the properties and applications of chitosan, cellulose and deep eutectic solvent in green chemistry. J. Ind. Eng. Chem..

[11] Aranaz, Alcántara A.R., Civera M.C., Arias C., Elorza B., Caballero A.H., Acosta N. (2021). Chitosan: An Overview of Its Properties and Applications. Polymers.

[12] Rahman L., Goswami J. (2021). Recent development on physical and biological properties of chitosan-based composite films with natural extracts: A review. J. Bioact. Compat. Polym..

[13] Kmiec M., Pighinelli L., Tedesco M.F., Silva M.M., Reis V. (2017). Chitosan-properties and applications in dentistry. Adv. Tissue Eng. Regen. Med..

[14] Bravo-Anaya L.M., Fernández-Solís K.G., Rosselgong J., Nano-Rodríguez J.L.E., Carvajal F., Rinaudo M. (2019). Chitosan-DNA polyelectrolyte complex: Influence of chitosan characteristics and mechanism of complex formation. Int. J. Biol. Macromol..

[15] Cao Y., Tan Y.F., Wong Y.S., Liew M.W.J., Venkatraman S. (2019). Recent Advances in Chitosan-Based Carriers for Gene Delivery. Mar. Drugs.

[16] Hashem F.M., Nasr M., Khairy A., Alqurshi A. (2019). In vitro cytotoxicity and transfection efficiency of pDNA encoded p53 gene-loaded chitosan-sodium deoxycholate nanoparticles. Int. J. Nanomed..

[17] Ahmed I., Dildar L., Haque A., Patra P., Mukhopadhyay M., Hazra S., Kulkarni M., Thomas S., Plaisier J.R., Dutta S.B. (2018). Chitosan-fatty acid interaction mediated growth of Langmuir monolayer and Langmuir-Blodgett films. J. Colloid Interface Sci..

[18] Li H., Zhang Z., Bao X., Xu G., Yao P. (2018). Fatty acid and quaternary ammonium modified chitosan nanoparticles for insulin delivery. Colloids Surf. B Biointerfaces.

[19] Lamptey R.N.L., Gothwal A., Trivedi R., Arora S., Singh J. (2022). Synthesis and Characterization of Fatty Acid Grafted Chitosan Polymeric Micelles for Improved Gene Delivery of VGF to the Brain through Intranasal Route. Biomedicines.

[20] Martins G.O., Petrônio M.S., Lima A.M.F., Junior A.M.M., Tiera V.A.D., Calmon M.d., Vilamaior P.S.L., Han S.W., Tiera M.J. (2019). Amphipathic chitosans improve the physicochemical properties of siRNA-chitosan nanoparticles at physiological conditions. Carbohydr. Polym..

[21] Gupta A., Pal A.K., Woo E.M., Katiyar V. (2018). Effects of Amphiphilic Chitosan on Stereocomplexation and Properties of Poly(lactic acid) Nano-biocomposite. Sci. Rep..

[22] Ali A., Ahmed S. (2018). A review on chitosan and its nanocomposites in drug delivery. Int. J. Biol. Macromol..

[23] Parhi R. (2020). Drug delivery applications of chitin and chitosan: A review. Environ. Chem. Lett..

[24] Naskar S., Kuotsu K., Sharma S. (2019). Chitosan-based nanoparticles as drug delivery systems: A review on two decades of research. J. Drug Target.

[25] Chang P.-H., Chao H.-M., Chern E., Hsu S.-H. (2021). Chitosan 3D cell culture system promotes naïve-like features of human induced pluripotent stem cells: A novel tool to sustain pluripotency and facilitate differentiation. Biomaterials.

[26] Ways T.M.M., Lau W.M., Khutoryanskiy V.V. (2018). Chitosan and Its Derivatives for Application in Mucoadhesive Drug Delivery Systems. Polymers.

[27] Lepeltier E., Loretz B., Desmaele D., Zapp J., Herrmann J., Couvreur P., Lehr C.-M. (2018). Squalenoylation of Chitosan: A Platform for Drug Delivery?. Biomacromolecules.

[28] Steinle H., Ionescu T.-M., Schenk S., Golombek S., Kunnakattu S.-J., Özbek M.T., Schlensak C., Wendel H.P., Avci-Adali M. (2018). Incorporation of Synthetic mRNA in Injectable Chitosan-Alginate Hybrid Hydrogels for Local and Sustained Expression of Exogenous Proteins in Cells. Int. J. Mol. Sci..

[29] Kyzas G.Z., Bikiaris D.N., Mitropoulos A.C. (2017). Chitosan adsorbents for dye removal: A review. Polym. Int..

[30] Shin J.-H., Yang J.E., Park J.E., Jeong S.-W., Choi S.-J., Choi Y.J., Jeon J. (2022). Rapid and Efficient Removal of Anionic Dye in Water Using a Chitosan-Coated Iron Oxide-Immobilized Polyvinylidene Fluoride Membrane. ACS Omega.

[31] Verma S., Dutta R.K., Naushad M., Lichtfouse E. (2020). Adsorptive Removal of Toxic Dyes Using Chitosan and Its Composites. Green Materials for Wastewater Treatment. Environmental Chemistry for a Sustainable World.

[32] Zhang Y., Zhao M., Cheng Q., Wang C., Li H., Han X., Fan Z., Su G., Pan D., Li Z. (2021). Research progress of adsorption and removal of heavy metals by chitosan and its derivatives: A review. Chemosphere.

[33] Borgohain R., Pattnaik U., Prasad B., Mandal B. (2021). A review on chitosan-based membranes for sustainable CO_2_ separation applications: Mechanism, issues, and the way forward. Carbohydr. Polym..

[34] Rosli N.A.H., Loh K.S., Wong W.Y., Yunus R.M., Lee T.K., Ahmad A., Chong S.T. (2020). Review of Chitosan-Based Polymers as Proton Exchange Membranes and Roles of Chitosan-Supported Ionic Liquids. Int. J. Mol. Sci..

[35] Dudek G., Gnus M., Turczyn R., Strzelewicz A., Krasowska M. (2014). Pervaporation with chitosan membranes containing iron oxide nanoparticles. Sep. Purif. Technol..

[36] Castro-Muñoz R., González-Valdez J., Zamidi A.M. (2021). High-performance pervaporation chitosan-based membranes: New insights and perspectives. Rev. Chem. Eng..

[37] Long Q., Zhang Z., Qi G., Wang Z., Chen Y., Liu Z.-Q. (2020). Fabrication of Chitosan Nanofiltration Membranes by the Film Casting Strategy for Effective Removal of Dyes/Salts in Textile Wastewater. ACS Sustain. Chem. Eng..

[38] Cazón P., Vázquez M., Crini G., Lichtfouse E. (2017). Applications of Chitosan as Food Packaging Materials. Sustainable Agriculture Reviews.

[39] Souza V.G.L., Pires J.R.A., Rodrigues C., Coelhoso I.M., Fernando A.L. (2020). Chitosan Composites in Packaging Industry—Current Trends and Future Challenges. Polymers.

[40] Janik W., Wojtala A., Pietruszka A., Dudek G., Sabura E. (2021). Environmentally Friendly Melt-Processed Chitosan/Starch Composites Modified with PVA and Lignin. Polymers.

[41] Janik W., Nowotarski M., Shyntum D.Y., Banaś A., Krukiewicz K., Kudła S., Dudek G. (2022). Antibacterial and Biodegradable Polysaccharide-Based Films for Food Packaging Applications: Comparative Study. Materials.

[42] Croisier F., Jérôme C. (2013). Chitosan-based biomaterials for tissue engineering. Eur. Polym. J..

[43] Mahmodi G., Zarrintaj P., Taghizadeh A., Taghizadeh M., Manouchehri S., Dangwal S., Ronte A., Ganjali M.R., Ramsey J.D., Kim S.-J. (2020). From microporous to mesoporous mineral frameworks: An alliance between zeolite and chitosan. Carbohydr. Res..

[44] Taghizadeh M., Taghizadeh A., Yazdi M.K., Zarrintaj P., Stadler F.J., Ramsey J.D., Habibzadeh S., Rad S.H., Naderi G., Saeb M.R. (2022). Chitosan-based inks for 3D printing and bioprinting. Green Chem..

[45] Gaidhani K.A., Harwalker M., Bhambere D., Nirgude P.S. (2015). Lyophilization/Freeze Drying—A Review. World J. Pharm. Res..

[46] Svagan J., Jensen P., Dvinskikh S.V., Furó I., Berglund L.A. (2010). Towards tailored hierarchical in cellulose nanocomposite foams prepared by freezing/freeze drying. J. Mater. Chem..

[47] Borisova A., de Bruyn M., Budarin V.L., Shuttleworth P.S., Dodson J.R., Segatto M.L., Clark J.H. (2015). A sustainable freeze-drying route to porous polysaccharides with tailored hierarchical meso-and microporosity. Macromol. Rapid Commun..

[48] Tang X.C., Pikal M.J. (2004). Design of Freeze-Drying Processes for Pharmaceuticals: Practical Advice. Pharm. Res..

[49] Pisano R., Fissore D., Velardi S.A., Barresi A.A. (2010). In-Line Optimization and Control of an Industrial Freeze-Drying Process for Pharmaceuticals. J. Pharm. Sci..

[50] Sadikoglu H., Ozdemir M., Seker M. (2006). Freeze-Drying of Pharmaceutical Products: Research and Development Needs. Dry. Technol..

[51] Ratti C., Bhandari B., Bansal N., Zhang M., Schuck P. (2013). 3—Freeze drying for food powder production. Handbook of Food Powders.

[52] Oikonomopoulou V.P., Krokida M.K., Karathanos V.T. (2011). The influence of freeze drying conditions on microstructural changes of food products. Procedia Food Sci..

[53] Jingjing E., Chen J., Chen Z., Ma R., Zhang J., Yao C., Wang R., Zhang Q., Yang Y., Li J. (2021). Effects of different initial pH values on freeze-drying resistance of Lactiplantibacillus plantarum LIP-1 based on transcriptomics and proteomics. Food Res. Int..

[54] Claussen I.A., Ustad T.S., Strommen I., Walde P.M. (2007). Atmospheric Freeze Drying—A Review. Dry. Technol..

[55] Oyinloye T.M., Yoon W.B. (2020). Effect of Freeze Drying on Quality and Grinding Process of Food Produce: A Review. Processes.

[56] Khan M.I.H., Wellard R.M., Nagy S.A., Joardder M.U.H., Karim M.A. (2017). Experimental investigation of bound and free water transport process during drying of hygroscopic food material. Int. J. Therm. Sci..

[57] Garg T., Chanana A., Joshi R. (2012). Preparation of Chitosan Scaffolds for Tissue Engineering using Freeze Drying Technology. IOSR J. Pharm..

[58] Song W., Xu J., Gao L., Zhang Q., Tong J., Ren L. (2021). Preparation of Freeze-Dried Porous Chitosan Microspheres for the Removal of Hexavalent Chromium. Appl. Sci..

[59] Tomaz A.F., de Carvalho S.M.S., Barbosa M.C., Silva S.M.L., Gutierrez M.A.S., de Lima A.G.B., Fook M.V.L. (2018). Ionically Crosslinked Chitosan Membranes Used as Drug Carriers for Cancer Therapy Application. Materials.

[60] Zeng M., Yuan X., Yang Z., Qi C. (2014). Novel macroporous palladium cation crosslinked chitosan membranes for heterogeneous catalysis application. Int. J. Biol. Macromol..

[61] Madihally S.V., Matthew H.W.T. (1999). Porous chitosan scaffolds for tissue engineering. Biomaterials.

[62] Riniki K., Dutta P.K. (2010). Chitosan based scaffolds by lyophilization and sc. CO_2_ assisted methods for tissue engineering applications. J. Macromol. Sci..

[63] Wu Y., Ma H., Wang J., Qu W. (2021). Production of chitosan scaffolds by lyophilization or electrospinning; which is better for peripheral nerve regeneration?. Neural Regen. Res..

[64] Ji C., Shi J. (2013). Thermal-crosslinked porous chitosan scaffolds for soft tissue engineering applications. Mater. Sci. Eng. C.

[65] Silvestro I., Sergi R., D’Abusco A.S., Mariano A., Martinelli A., Piozzi A., Francolini I. (2021). Chitosan scaffolds with enhanced mechanical strength and elastic response by combination of freeze gelation, photo-crosslinking and freeze-drying. Carbohydr. Polym..

[66] Qian L., Zhang H. (2013). One-step synthesis of protein-encapsulated microspheres in a porous scaffolds by freeze-drying double emulsions and tuneble protein release. Chem. Commun..

[67] Yang B., Li X.Y., Shi S., Kong X.Y., Guo G., Huang M.J., Luo F., Wei Y.Q., Zhao X., Qian Z.Y. (2010). Preparation and characterization of a novel chitosan scaffold. Carbohydr. Polym..

[68] Seol Y.-J., Lee J.-Y., Park Y.-J., Lee Y.-M., Ku Y., Rhyu I.-C., Lee S.-J., Han S.-B., Chung C.-P. (2004). Chitosan sponges as tissue engineering scaffolds for bone formation. Biotechnol. Lett..

[69] Kim S.E., Park J.H., Cho Y.W., Chung H., Jeong S.Y., Lee E.B., Kwon I.C. (2003). Porous chitosan scaffold containing microspheres loaded with transforming growth factor-β1: Implications for cartilage tissue engineering. J. Control. Release.

[70] Heimbuck M., Priddy-Arrington T.R., Sawyer B.J., Caldorera-Moore M.E. (2019). Effects of post-processing methods on chitosan-genipin hydrogel properties. Mater. Sci. Eng. C.

[71] Bharadwaz A., Jayasuriya A.C. (2021). Fabrication of porous chitosan particles using a novel two-step porogen leaching and lyophilization method with the label-free multivariate spectral assessment of live adhered cells. Colloids Surf. B.

[72] Qian L., Zhang H. (2010). Green synthesis of chitosan-based nanofibers and their applications. Green Chem..

[73] Lu Z., Zhou Y., Liu B. (2019). Preparation of chitosan microcarriers by high voltage electrostatic field and freeze drying. J. Biosci. Bioeng..

[74] Ren L., Xu J., Zhang Y., Zhou J., Chen D., Chang Z. (2019). Preparation and characterization of porous chitosan microspheres and adsorption performance for hexavalent chromium. Int. J. Biol. Macromol..

[75] Guilak F., Cohen D.M., Estes B.T., Gimble J.M., Liedtke W., Chen C.S. (2009). Control of Stem Cell Fate by Physical Interactions with the Extracellular Matrix. Cell Stem Cell.

[76] Zhu C., Huang J., Xue C., Wang Y., Wang S., Bao S., Chen R., Li Y., Gu Y. (2018). Skin derived precursor Schwann cell-generated acellular matrix modified chitosan/silk scaffolds for bridging rat sciatic nerve gap. Neurosci. Res..

[77] Gu Y., Ji Y., Zhao Y., Liu Y., Ding F., Gu X., Yang Y. (2012). The influence of substrate stiffness on the behavior and functions of Schwann cells in culture. Biomaterials.

[78] Benhabbour S.R., Sheardown H., Adronov A. (2008). Cell adhesion and proliferation on hydrophilic dendritically modified surfaces. Biomaterials.

[79] Ranganathan S., Balagangadharan K., Selvamurugan N. (2019). Chitosan and gelatin-based electrospun fibers for bone tissue engineering. Int. J. Biol. Macromol..

[80] Chen Y., Qi Y., Yan X., Ma H., Chen J., Liu B., Xue Q. (2014). Green fabrication of porous chitosan/graphene oxide xerogels for drug delivery. J. Appl. Surf. Sci..

[81] Lozinsky V.I. (2018). Cryostructuring of Polymeric Systems. 50. † Cryogels and Cryotropic Gel-Formation: Terms and Definitions. Gels.

[82] Lozinsky V.I., Okay O. (2014). Basic Principles of Cryotropic Gelation. Adv. Polym. Sci..

[83] Santos-López G., Argüelles-Monal W., Carvajal-Millan E., López-Franco Y.L., Recillas-Mota M.T., Lizardi-Mendoza J. (2017). Aerogels from Chitosan Solutions in Ionic Liquids. Polymers.

[84] Nielsen N.E. (1969). Cross-linking-effect on physical properties of polymers. Polym. Rev..

[85] Rabek J.F. (2009). Contemporary Knowledge about Polymers.

[86] Ho M.-H., Kuo P.-Y., Hsieh H.-J., Hsien T.-Y., Hou L.-T., Lai J.-Y., Wang D.-M. (2004). Preparation of porous scaffolds by using freeze-extraction and freeze-gelation methods. Biomaterials.

[87] Chen P.H., Hwang Y.-H., Kuo T.-Y., Liu F.-H., Lai J.-Y., Hsieh H.-J. (2007). Improvement in the Properties of Chitosan Membranes Using Natural Organic Acid Solutions as Solvents for Chitosan Dissolution. J. Med. Bioeng..

[88] Pinto R.V., Gomes P.S., Fernandes M.H., Costa M.E.V., Almeida M.M. (2020). Glutaraldehyde-crosslinking chitosan scaffolds reinforced with calcium phosphate spray-dried granules for bone tissue applications. Mater. Sci. Eng. C.

[89] Pierre A.C. (2020). Introduction to Sol-Gel Processing.

[90] Parashar M., Shukla V., Singh R. (2020). Metal oxides nanoparticles via sol–gel method: A review on synthesis, characterization and applications. J. Mater. Sci. Mater. Electron..

[91] Cividanes L.S., Campos T.M.B., Rodrigues L.A., Brunelli D.D., Thim G.P. (2010). Review of mullite synthesis routes by sol–gel method. J. Sol-Gel Sci. Technol..

[92] Maleki H., Durães L., García-González C.A., del Gaudio P., Portugal A., Mahmoudi M. (2016). Synthesis and biomedical applications of aerogels: Possibilities and challenges. Adv. Colloid Interface Sci..

[93] Montembault A., Viton C., Domard A. (2005). Rheometric Study of the Gelation of Chitosan in Aqueous Solution without Cross-Linking Agent. Biomacromolecules.

[94] Dobashi T., Tomita N., Maki Y., Chang C.P., Yamamoto T. (2011). An analysis of anisotropic gel forming process of chitosan. Carbohydr. Polym..

[95] Cui Z., Xiang Y., Si J., Yang M., Zhang Q., Zhang T. (2008). Ionic interactions between sulfuric acid and chitosan membranes. Carbohydr. Polym..

[96] Fan W., Yan W., Xu Z., Ni H. (2012). Formation mechanism of monodisperse, low molecular weight chitosan nanoparticles by ionic gelation technique. Colloids Surf. B.

[97] Priyadarshi R., Sauraj, Kumar B., Negi Y.S. (2018). Chitosan film incorporated with citric acid and glycerol as an active packaging material for extension of green chilli shelf life. Carbohydr. Polym..

[98] Hamdine M., Heuzey M.-C., Bégin A. (2006). Viscoelastic properties of phosphoric and oxalic acid-based chitosan hydrogels. Rheol. Acta.

[99] Beppu M.M., Vieira R.S., Aimoli C.G., Santana C.C. (2007). Crosslinking of chitosan membranes using glutaraldehyde: Effect on ion permeability and water absorption. J. Membr. Sci..

[100] Medellín-Castillo N.A., Isaacs-Páez E.D., Rodríguez-Méndez I., González-García R., Labrada-Delgado G.J., Aragón-Piña A., García-Arreola M.E. (2021). Formaldehyde and tripolyphosphate crosslinked chitosan hydrogels: Synthesis, characterization and modelling. Int. J. Biol. Macromol..

[101] Chen A.-H., Liu S., Chen C., Chen C.-Y. (2008). Comparative adsorption of Cu(II), Zn(II), and Pb(II) ions in aqueous solution on the crosslinked chitosan with epichlorohydrin. J. Hazard. Mater..

[102] Muzzarelli R.A. (2009). Genipin-crosslinked chitosan hydrogels as biomedical and pharmaceutical aids. Carbohydr. Polym..

[103] Yang Q., Dou F., Liang B., Shen Q. (2005). Studies of cross-linking reaction on chitosan fiber with glyoxal. Carbohydr. Polym..

[104] Stievano M., Elvassore N. (2005). High-pressure density and vapor–liquid equilibrium for the binary systems carbon dioxide–ethanol, carbon dioxide–acetone and carbon dioxide–dichloromethane. J. Supercrit. Fluids.

[105] Wei S., Ching Y.C., Chuah C.H. (2020). Synthesis of chitosan aerogels as promising carriers for drug delivery: A review. Carbohydr. Polym..

[106] López-Iglesias C., Barros J., Ardao I., Monteiro F., Alvarez-Lorenzo C., Gómez-Amoza J., García-González C. (2019). Vancomycin-loaded chitosan aerogel particles for chronic wound applications. Carbohydr. Polym..

[107] Zhang S., Feng J., Feng J., Jiang Y. (2017). Formation of enhanced gelatum using ethanol/water binary medium for fabricating chitosan aerogels with high specific surface area. Chem. Eng. J..

[108] Takeshita S., Yoda S. (2015). Chitosan aerogels: Transparent flexible thermal insulators. Chem. Mater..

[109] Takeshita S., Akasaka S., Yoda S. (2019). Structural and acoustic properties of transparent chitosan aerogel. Mater. Lett..

[110] Obaidat R.M., Tashtoush B.M., Bayan M., Al Bustami R.T., Alnaief M. (2015). Drying Using Supercritical Fluid Technology as a Potential Method for Preparation of Chitosan Aerogel Microparticles. Pharm. Sci. Tech..

[111] Takeshita S., Konishi A., Takebayashi Y., Yoda S., Otake K. (2017). Aldehyde Approach to Hydrophobic Modification of Chitosan Aerogels. Biomacromolecules.

[112] Takeshita S., Zhao S., Malfait W.J. (2021). Transparent, Aldehyde-Free Chitosan Aerogel. Carbohydr. Polym..

[113] Venault A., Chang Y., Wang D.-M., Bouyer D. (2013). A Review on Polymeric Membranes and Hydrogels Prepared by Vapor-Induced Phase Separation Process. Polym. Rev..

[114] Hołda A.K., Vankelecom I.F.J. (2015). Understanding and guiding the phase inversion process for synthesis of solvent resistant nanofiltration membranes. J. Appl. Polym. Sci..

[115] Matsuyama H., Karkhanechi H., Rajabzadeh S., Chung T.-S., Feng Y. (2021). Chapter 3—Polymeric membrane fabrication via thermally induced phase separation (TIPS) method. Hollow Fiber Membranes.

[116] Mi F.-L., Wu Y.-B., Shyu S.-S., Chao A.-C., Lai J.-Y., Su C.-C. (2003). Asymmetric chitosan membranes prepared by dry/wet phase separation: A new type of wound dressing for controlled antibacterial release. J. Membr. Sci..

[117] Hong H., Wei J., Liu C. (2007). Development of asymmetric gradational-changed porous chitosan membrane for guided periodontal tissue regeneration. Compos. Part B.

[118] Gu Z.Y., Xue P.H., Li W.J. (2001). Preparation of the Porous Chitosan Membrane by Cryogenic Induced Phase Separation. Polym. Adv. Technol..

[119] Qin W., Li J., Tu J., Yang H., Chen Q., Liu H. (2017). Fabrication of porous chitosan membranes composed of nanofibers by low temperature thermally induced phase separation, and their adsorption behavior for Cu^2+^. Carbohydr. Polym..

[120] Mi F.-L., Shyu S.-S., Chen C.-T., Schoung J.-Y. (1999). Porous chitosan microsphere for controlling the antigen release of Newcastle disease vaccine: Preparation of antigen-adsorbed microsphere and in vitro release. Biomaterials.

[121] Salerno S., de Santo M.P., Drioli E., de Bartolo L. (2021). Nano-and Micro-Porous Chitosan Membranes for Human Epidermal Stratification and Differentiation. Membranes.

[122] Biswas D.P., Tran P.A., Tallon C., O’Connor A.J. (2017). Combining mechanical foaming and thermally induced phase separation to generate chitosan scaffolds for soft tissue engineering. J. Biomater. Sci. Polym. Ed..

[123] Mansour F.R., Waheed S., Paull B., Maya F. (2020). Porogens and porogen selection in the preparation of porouspolymer monoliths. J. Sep. Sci..

[124] Chevalier E., Chulia D., Pouget C., Viana M. (2008). Fabrication of Porous Substrates: A Review of Processes Using Pore Forming Agents in the Biomaterial Field. J. Pharm. Sci..

[125] Janik H., Marzec M. (2015). A review: Fabrication of porous polyurethane scaffolds. Mater. Sci. Eng. C Mater. Biol. Appl..

[126] Zhou M., Shen L., Lin X., Hong Y., Feng Y. (2017). Design and pharmaceutical applications of porous particles. RSC Adv..

[127] Chao A.-C., Yu S.-H., Chuang G.-S. (2006). Using NaCl particles as porogen to prepare a highly adsorbent chitosan membranes. J. Membr. Sci..

[128] Santos D.E.S., Neto C.G.T., Fonseca J.L.C., Pereira M.R. (2008). Chitosan macroporous asymmetric membranes—Preparation, characterization and transport of drugs. J. Membr. Sci..

[129] Zeng M., Fang Z., Xu C. (2004). Novel method of preparing microporous membrane by selective dissolution of chitosan/polyethylene glycol blend membrane. J. Appl. Polym. Sci..

[130] Lim J.I., Lee Y.-K., Shin J.-S., Lim K.-J. (2011). Preparation of interconnected porous chitosan scaffolds by sodium acetate particulate leaching. J. Biomater. Sci. Polym. Ed..

[131] Liu Y., Cai Z., Ma M., Sheng L., Huang X. (2020). Effect of eggshell membrane as porogen on the physicochemical structure and protease immobilization of chitosan-based macroparticles. Carbohydr. Polym..

[132] Shi C., Lv C., Wu L., Hou X. (2017). Porous chitosan/hydroxyapatite composite membrane for dyes static and dynamic removal from aqueous solution. J. Hazard. Mater..

[133] Rekik S.B., Gassara S., Bouaziz J., Deratani A., Baklouti S. (2019). Enhancing hydrophilicity and permeation flux of chitosan/kaolin composite membranes by using polyethylene glycol as porogen. Appl. Clay Sci..

[134] Alias S.S., Ariff Z.M., Mohamad A.A. (2015). Porous membrane based on chitosan–SiO2 for coin cell proton battery. Ceram. Int..

